# Leadership styles and leadership outcomes in nursing homes: a cross-sectional analysis

**DOI:** 10.1186/s12913-020-05854-7

**Published:** 2020-11-04

**Authors:** Joris Poels, Marc Verschueren, Koen Milisen, Ellen Vlaeyen

**Affiliations:** 1 Department of Public Health and Primary Care, Academic Centre for Nursing and Midwifery, KU Leuven, Kapucijnenvoer 35, 4th floor, P.B. 7001, 3000 Leuven, Belgium; 2 Development & Education, University Hospitals Leuven, Leuven, Belgium; 3 Department of Internal Medicine, Division of Geriatric Medicine, University Hospitals Leuven, Leuven, Belgium

**Keywords:** Leadership, Residential care facilities, Nursing home, Care manager, Nurse, Director of nursing

## Abstract

**Background:**

Although leadership is considered as a key factor in health care, leadership styles and outcomes in nursing homes often remain a black box. Therefore, this study explored leadership styles and leadership outcomes of head nurses and directors of nursing (DoN) in nursing homes based on well-defined leadership concepts.

**Methods:**

A multicenter cross-sectional analysis was conducted on baseline data of an ongoing cohort study comprising a convenience sample of nursing home staff (*n* = 302). Leadership styles and leadership outcomes of head nurses and DoN were measured through the rater form of the Multifactor Leadership Questionnaire 5X (MLQ-5X). Based on the Full Range of Leadership Model, the MLQ-5X visualizes transformational (relation and change focused), transactional (task-focused) and passive-avoidant (absence of leadership) leadership styles. Scores of head nurses and DoN for leadership styles and outcomes were compared with European Reference Scores (ERS) using two-sided one-sample t-tests.

**Results:**

Compared with ERS, head nurses and DoN scored significantly lower (*p* < 0.001) on transformational and transactional leadership styles and significantly higher (*p* < 0.001) on passive-avoidant leadership styles. All leadership outcomes were significantly lower (*p* < 0.001) for head nurses. Similar results, however not statistically significant, were found concerning leadership outcomes of DoN.

**Conclusions:**

Results indicate that passive-avoidant leadership styles are excessively present in contrast to transformational leadership styles in nursing homes. This highlights an urgent need to invest in leadership development. Therefore, future research should focus on interventions for the development of transformational leadership.

## Background

Worldwide demographic evolutions affect the organization of health care. Especially nursing homes face daunting challenges to meet present and future care needs [1, 2]. Firstly, individuals consider a nursing home as a last resort and therefore prefer to live as long as possible in their own home [3]. Consequently, most residents admitted to a nursing home suffer from multiple complex conditions and are in need of more assistance [4, 5]. Secondly, poor work organization, time pressure, high workload, staff shortages and turnover are potential threats to quality of care and patient safety [6–9]. Previous research showed high annualized turnover rates for certified nurse assistants (74.5%), registered nurses (56.1%) and licensed practical nurses (51.0%) in nursing homes [10]. The American Association of Colleges of Nursing predicts by 2025, without appropriate intervention strategies, a shortage of approximately 260,000 registered nurses in the United States [11]. These shortages could negatively affect the number of staff available in nursing homes. Moreover, the Institute of Medicine reports financial and work environment related challenges, such as low wages and job dissatisfaction, in the recruitment and retention of direct-care workers [12]. In addition, both intention to leave and to stay among nurses are associated with leadership practices [13]. In general, literature increasingly describes leadership as a key factor in solving challenges in health care. Northouse (2013) defines leadership as ‘a process whereby an individual influences a group of people in order to achieve a common goal’. Its contribution is considered equally important in enhancing the quality of care as in a complex and high risk environment like aviation [14, 15]. For example, when staff perceives leadership as good it positively influences their retention and job satisfaction [13, 16].

Leaders can exhibit a variety of leadership styles, characterized by behavioral dimensions. Task-oriented leadership focuses on coordinating and assigning work to followers. Change-oriented leadership aims at the identification, envisioning and managing of change on team level. Relation-oriented leadership comprises of team development and support [17, 18]. In particular, the Full Range of Leadership Model (FRLM) of Avolio and Bass provides a broad perspective on leadership styles. The first leadership style, *transformational leadership,* motivates followers to do more than what is expected of them. This leadership style aims to increase the levels of motivation and morality among followers, by invoking *idealized influence, individualized consideration, inspirational motivation* and*. Intellectual stimulation.* Firstly, *Idealized influence* comprises two subcomponents: 1) *Idealized influence attributed* describes the extent to which a leader works on trusting relationships, whereas 2) *idealized influence behavior* focuses on the degree to which a leader acts with integrity and works on a collective mission. Secondly, *individual consideration* measures the extent to which a leader supports and coaches group members. Thirdly, *inspirational motivation* is measured to gain insight in the extent to which a leader motivates followers with an inspiring vision. Fourthly, *intellectual stimulation* measures the degree that a leader appeals on the abilities of employees to identify problems and to approach these problems creatively. Transformational leadership will often result in performance that surpasses the expected outcomes [19]. The second leadership style, *transactional leadership,* emphasizes the exchange relationship between leader and follower; both encouraged to meet their own needs. Transactional leadership has two components: 1) *contingent reward* refers to clarifying roles and tasks, and providing followers with material or psychological rewards contingent on the fulfillment of obligations; 2) *active management by exception* refers to a leader actively monitoring the work of followers so that, in case of errors, corrective actions can be undertaken. Transactional leadership will often result in expected outcomes [19]. Finally, the third leadership style, passive-avoidant leadership, consists of two components: 1) *passive management-by-exception*, reflecting avoidance of leadership, and 2) laissez-faire, which means absence of leadership [15, 20]. In addition to measuring leadership styles, the FRLM provides a questionnaire (the MLQ-5X) that also includes nine questions on three outcomes of leadership behavior. The first leadership outcome, *extra effort,* measures how often followers perceive their leader as someone that motivates others to do more, heightens desires to succeed and increases willingness to try harder. The second, *effectiveness,* reflects how successful a leader interacts at different levels of the organization, representing a group to higher authorities and meeting others’ job-related needs. The third, *satisfaction,* measures whether followers are satisfied with their leader’s working methods [21].

The most recommended style of relation-oriented leadership, included in the FRLM, is transformational leadership [17]. Focusing on the development and implementation of change, this leadership style motivates followers to perform beyond what is expected of them [19, 22]. Literature suggests a link between transformational leadership and several factors. In terms of workforce outcomes, transformational leadership is associated with increased staff-wellbeing, higher job satisfaction, decreased intention to leave and decreased burn-out rate [23–25]. One study shows a direct negative relation between burn-out and transformational leadership (β = − 0.19, *p* < 0.01) [26]. This leadership style also positively correlates with patient outcomes such as higher patient satisfaction, higher quality of care, lower mortality and less medication errors [27, 28]. Lastly, transformational leadership relates to organizational outcomes such as increased innovation capacity [29]. Although the potential of transformational leadership in health care is well described, the empiric literature on leadership styles in nursing homes often remains conceptually unclear. Apart from a few studies that suggest the importance of relation-oriented leadership, the leadership styles measured in nursing homes frequently are a black box to be unravelled [30, 31]. Given the need of transformational leadership as an indispensable element in developing, implementing and sustaining the crucial changes that health care needs to make to improve quality of care and patient safety, this study aims to explore leadership styles in nursing homes based on well-described concepts [32, 33]. In addition, this study will also explore outcomes of the present leadership styles.

## Methods

This study was reported following the STROBE guidelines for observational studies [34].

### Sample, setting and design

A cross-sectional analysis was conducted on baseline data of an ongoing cohort study in 2015 comprising a convenience sample of staff in six nursing homes in Belgium. Nursing homes were included if the managing board agreed to participate, but were excluded if they already participated in another study. Participating staff had to speak and read Dutch. The Medical Ethics Committee of the University Hospitals Leuven approved this study with EC number S52526. All participating staff provided informed consent by voluntarily completing the data collection.

### Procedure

**Fig. 1:**
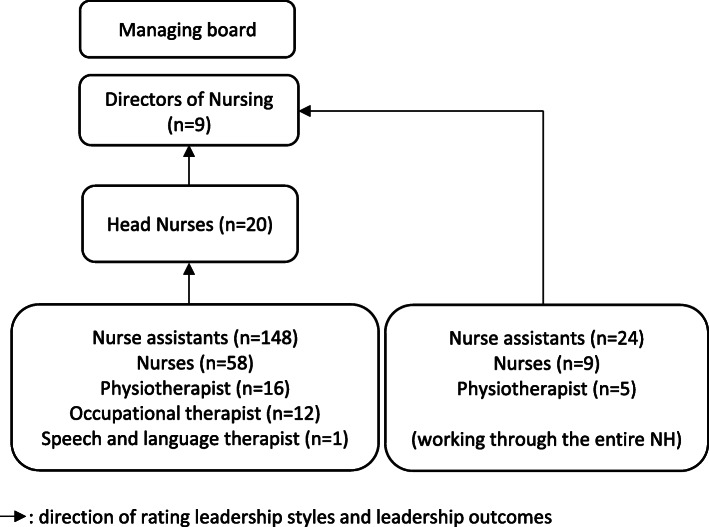
Rating of leadership styles and leadership outcomes by staff in nursing home: direction of rating leadership styles and leadership outcomes

In every nursing home, a moment was scheduled in which staff completed a survey, rating leadership styles of their direct supervisor. Two levels of leadership were examined (Fig. 1). The first level involved the leadership of head nurses, who plan, coordinate, monitor, control and adjust the activities on their ward in order to ensure that staff can deliver individualized care in optimal conditions [35, 36]. The second level involved the leadership of directors of nursing (DoN), who are continuously balancing between clinical leadership and managerial targets. DoN have a complex role in budgets and finances, staff recruitment, handling staff conflicts, monitoring of care quality and representation to other organizations [37]. Head nurses were rated by at least five staff members working on their ward. DoN were rated by all head nurses in the respective nursing home. Some staff reported to work throughout the entire nursing home and did not solely belong to one ward. They report directly to the DoN and therefore rated the DoN as their direct supervisor. Participants had to master Dutch and participate voluntarily.

### Measures

A validated Dutch version of the Multifactor Leadership Questionnaire 5X (MLQ-5X) form was used to measure leadership styles and outcomes of head nurses and DoN [21]. The MLQ-5X comprises 45 items divided into nine subscales to capture a broad range of leadership behaviors. Items are scored using a five-point Likert scale with responses that range from “never” (0) to “frequently, if not always” [4]. They reflect the degree to which certain leadership behaviors are present and are based on the components of the Full Range of Leadership Model (FRLM) of Avolio and Bass [21]. The FRLM is a well-established model, supported by the accumulation of evidence on its validity throughout 30 years [38]. Transformational, transactional and passive avoidant leadership styles, respectively ranked from higher to lower effectivity, are included in the model [19]. Previous research confirmed adequate construct and predictive validity of the MLQ-5X in that it satisfies the model fit requirement. In addition, based on factorial invariance tests to study the model’s consistency, previous research confirmed the reliability of the MLQ-5X in that it measures the same constructs across multiple groups [39]. To interpret the acquired data the MLQ-5X manual provides a scoring key that indicates which scale items should be grouped. Transformational, transactional and passive avoidant leadership are respectively measured by twenty, eight and eight items. For leadership outcomes, which can be considered as results of leadership behavior, extra effort, effectiveness and satisfaction are respectively measured by three, four and two items [21]. Because of copyright stipulations, more detailed information about the questionnaire, scoring key and which items are related to which leadership style is provided in the MLQ-5X Manual [21].

### Analysis

Analysis were performed using SPSS version 22.0 (IBM SPSS, Armonk, NY: IBM Corp.). Analysis were performed for leadership styles of head nurses and DoN in the overall sample. For each component of the FRLM means were calculated. Using a two-sided one-sample t-test, means were compared with the European Reference Scores (ERS), which are normative scores for leadership styles and outcomes based on cross-cultural research [21]. Scores of head nurses and DoN were compared to norm scores for their respective levels, based on normative samples of respectively 3061 and 1222 respondents with a high executive or senior staff function [21]. To correct for multiple testing, a Bonferroni-adjustment was used, setting the significance threshold at α = 0.002 [40]. A Pearson product-moment correlation coefficient was calculated for each FRLM-component. Cronbach’s alpha was used to test the internal consistency of the MLQ-5X subscales, considering a value of ≥0.80 as good and < 0.60 as poor [41].

## Results

### Head nurses

In total 242 staff received the questionnaires on leadership styles and outcomes of 22 head nurses of which 235 completed these questions (response rate: 97.1%). Respondents are mainly female (91.5%), with a mean age of 38.1 years (±12.1y) and work on average 10 years in the current nursing home (±10.1y). They work as nurse-assistant (63.0%), nurse (24.7%), physiotherapist (6.8%), occupational therapist (5.1%) and general practitioner (0.4%), with a mean number of 28.3 h (±10.6 h) per week. Table 1 shows that, compared with the European Reference Scores (ERS), staff rates their direct leaders significantly lower (*p* < 0.001) on four out of five components of transformational leadership: idealized influence attributed, idealized influence behavior, inspirational motivation, intellectual stimulation and individual consideration. Staff rates contingent reward, being part of transactional leadership, also significantly lower (*p* < 0.001) in head nurses, whereas passive-avoidant leadership scores are significantly higher (*p* < 0.001).

**Table 1:** Leadership styles of head nurses in nursing homes

Head nurses	Mean (±SD) (CI 99.8%)	ERS	Sample	Correlations								Cronbach’s alpha
				(1)	(2)	(3)	(4)	(5)	(6)	(7)	(8)	
**Transformational**												
**(1)** Idealized influence: attributed	2.17^**<**^ (±0.67) (2.04–2.31)	2.72	234									0.48
**(2)** Idealized influence: behavior	2.62 (±0.72) (2.48–2.77)	2.69	233	0.58**								0.74
**(3)** Inspirational motivation	2.62^**<**^ (±0.79) (2.45–2.78)	2.83	233	0.63**	0.80**							0.86
**(4)** Intellectual stimulation	2.56^**<**^ (±0.69) (2.42–2.70)	2.82	234	0.62**	0.77**	0.77**						0.78
**(5)** Individual consideration	2.42^**<**^ (±0.76) (2.27–2.58)	2.66	234	0.61**	0.65**	0.64**	0.65**					0.65
**Transactional**												
**(6)** Contingent Reward	2.57^**<**^ (±0.78) (2.41–2.73)	2.77	235	0.59**	0.79**	0.79**	0.76*	0.68**				0.81
**(7)** Management by exception: active	2.30 (±0.68) (2.16–2.44)	2.33	232	0.37**	0.33**	0.29**	0.25**	0.27**	0.30**			0.62
**Passive/avoidant**												
**(8)** Management by exception: passive	1.29^**>**^ (±0.71) (1.15–1.44)	1.10	233	−0.25**	− 0.34**	− 0.38**	− 0.36**	−0.23**	− 0.31**	0.05		0.54
**(9)** Laissez-faire	1.13^**>**^ (±0.78) (0.97–1.29)	0.79	234	−0.25**	−0.44**	− 0.46**	−0.44*	− 0.39**	−0.39**	0.07	0.65**	0.74

Scores on subscales (1–9) have an available range between 0 and 4; *CI* Confidence Interval, *ERS* European Reference Scores; ^<^: significant lower (*p* < 0.002) than ERS; ^>^: significant higher (*p* < 0.002) than ERS; *correlation significant (*p* < 0.05); **correlation significant (*p* < 0.01)

Staff scores head nurses significantly lower on all leadership outcomes (*p* < 0.001), compared with the ERS (see Table 2). Intercorrelations of MLQ-subscales are consistent with findings in the European normative sample.

**Table 2:** Leadership outcomes of head nurses in nursing homes

Head nurses	Mean (±SD) (CI 99.8%)	ERS	Sample	Correlations		Cronbach’s alpha
				(1)	(2)	
**(1)** Extra Effort	2.43^**<**^ (±0.79) (2.27–2.59)	2.75	231			0.79
**(2)** Effectiveness	2.64^**<**^ (±0.72) (2.49–2.79)	3.01	232	0.77**		0.84
**(3)** Satisfaction	2.67^**<**^ (±0.83) (2.50–2.84)	2.94	231	0.75**	0.83**	0.72

Scores on subscales (1–3) have an available range between 0 and 4; *CI* Confidence Interval; *ERS* European Reference Scores; ^**<**^: significant lower (*p* < 0.002) than ERS; ^**>**^: significant higher (*p* < 0.002) than ERS; *correlation significant (*p* < 0.05); **correlation significant (*p* < 0.01)

### Directors of nursing

Twenty head nurses and 38 other staff rated leadership styles and outcomes of 9 DoN (see Fig. 1). Respondents are mainly female (93.1%), with a mean age of 42.2 year (±9.3y) and work on average 11.8 years (±9.9y) in the current nursing home. They work as head nurse (34.5%), night nurse-assistant (41.4%), night nurse (15.5%) and physiotherapist (8.6%) with a mean number of 28.3 h (±10.6 h) per week. Table 3 shows that, compared with the ERS, DoN score significantly lower on idealized influence attributed and individual consideration (*p* < 0.001) whereas passive-avoidant leadership scores are significantly higher (p < 0.001).

**Table 3:** Leadership styles of directors of nursing in nursing homes

**Directors of Nursing**	Mean (±SD) (CI 99.8%)	ERS	Sample	Correlations								Cronbach’s alpha
				(1)	(2)	(3)	(4)	(5)	(6)	(7)	(8)	
**Transformational**												
**(1)** Idealized influence: attributed	2.36^**<**^ (±0.62) (2.10–2.63)	2.77	57									0.42
**(2)** Idealized influence: behavior	2.77 (±0.65) (2.49–3.04)	2.73	58	0.59**								0.74
**(3)** Inspirational motivation	2.85 (±0.66) (2.57–3.14)	2.68	57	0.67**	0.76**							0.86
**(4)** Intellectual stimulation	2.62 (±0.58) (2.37–2.87)	2.74	56	0.49**	0.68**	0.66**						0.73
**(5)** Individual consideration	2.31^**<**^ (±0.73) (1.99–2.62)	2.75	57	0.66**	0.50**	0.58**	0.48**					0.60
**Transactional**												
**(6)** Contingent Reward	2.77 (±0.69) (2.48–3.06)	2.90	58	0.66	0.64**	0.71**	0.71**	0.55**				0.75
**(7)** Management by exception: active	2.30 (±0.57) (2.05–2.54)	2.31	58	0.37**	0.39**	0.26	0.26	0.15	0.32*			0.06
**Passive/avoidant**												
**(8)** Management by exception: passive	1.56^**>**^ (±0.66) (1.28–1.84)	1.16	57	−0.31*	−0.23	−0.39**	−0.31*	−0.36**	−0.39**	0.19		0.56
**(9)** Laissez-faire	1.20^**>**^ (±0.77) (0.87–1.53)	0.85	58	−0.45**	−0.50**	− 0.56**	−0.43**	− 0.48**	−0.53**	0.02	0.55**	0.75

Scores on subscales (1–9) have an available range between 0 and 4; *CI* Confidence Interval, *ERS* European Reference Scores; ^<^: significant lower (*p* < 0.002) than ERS; ^>^: significant higher (*p* < 0.002) than ERS; *correlation significant (*p* < 0.05); **correlation significant (*p* < 0.01)

No significant results are found in outcomes of leadership in DoN (see Table 4). Intercorrelations of MLQ-subscales are consistent with findings in the European normative sample.

**Table 4:** Leadership outcomes of directors of nursing in nursing homes

Directors of Nursing	Mean (±SD) (CI 99.8%)	ERS	Sample	Correlations		Cronbach’s alpha
				(1)	(2)	
**(1)** Extra Effort	2.54 (±0.74) (2.22–2.86)	2.66	56			0.73
**(2)** Effectiveness	2.78 (±0.63) (2.51–3.05)	2.96	56	0.74**		0.76
**(3)** Satisfaction	2.86 (±0.74) (2.54–3.18)	2.92	57	0.69**	0.83**	0.86

Scores on subscales (1–3) have an available range between 0 and 4; *CI* Confidence Interval, *ERS* European Reference Scores; ^**<**^: significant lower (*p* < 0.002) than ERS; ^**>**^: significant higher (*p* < 0.002) than ERS; *correlation significant (*p* < 0.05); **correlation significant (*p* < 0.01)

### Reliability of the MLQ-5X

Cronbach’s alpha was used to test the reliability of the MLQ-5X subscales in both raters of head nurses and DoN (see Tables 1, 2, 3, and 4). With regard to raters of head nurses, internal consistency of scale items is poor for idealized influence attributed (α = 0.48) and passive management by exception (α = 0.54). In raters of DoN internal consistency is poor for idealized influence (α = 0.42), active (α = 0.06) and passive management by exception (α = 0.56). Cronbach’s alpha enhances only in the subscale *idealized influence attributed* if the item that considers transcending self-interest was deleted in both head nurses (α = 0.77) and DoN (α = 0.73).

## Discussion

To the best of our knowledge, this is the first study that quantitatively investigates and maps leadership styles and leadership outcomes of head nurses and directors of nursing (DoN) in nursing homes. Compared with the European Reference Scores (ERS), head nurses and DoN score significantly lower (*p* < 0.001) on components of transformational and transactional leadership, but score significantly higher (*p* < 0.001) on passive-avoidant leadership. In addition, head nurses score significantly lower than ERS on all leadership outcomes (*p* < 0.001). Similar results, however not statistically significant, are found concerning leadership outcomes of DoN. Previous research on leadership styles in nursing homes found that, based on the Bonoma-Slevin leadership model, an autocrat style was used by 25% of DoN. An autocrat leader does not involve, nor informs employees about decisions [42]. In addition, results showed that a consensus leadership style, that involves employees and encourages team decision making, was used by only 30% of DoN [42, 43]. Given the potential influences on care quality and patient safety, our results, showing low scores on transformational leadership styles and high scores on passive-avoidant leadership styles, are alarming. Several aspects may contribute to our study results. First, nursing leadership often seems to be conflated with administrative positions. Therefore, head nurses may not be able to lead staff if their available time is filled with administrative tasks that hinder a visible presence on their wards. Consequently, the low scores on leadership outcomes may reflect some dissatisfaction of staff with the current ‘absence’ and ‘avoidance’ of leadership [44]. Although leadership styles may be context dependent, previous research investigating characteristics of highly rated leadership confirmed non-avoidant and non-passive behaviors such as coaching, closely monitoring of work and giving direct feedback (i.e. being visible) as crucial in nursing home leaders [45]. Furthermore, this is also consistent with previous findings in hospitals, a leader that is perceived as ‘good’ by staff may be one that is visible [46]. Similarly, to effectively provide guidance to the nursing home managing board, DoN need to remain closely in contact with staff. Their recommendations should include issues and perspectives of staff (e.g. head nurses) ‘on the front line’ [47]. Second, there could be a discrepancy between the expected leadership and the perceived leadership of staff. Expectations of leadership are influenced by various factors, like for example education. Nurse-assistants and head nurses often have a different educational background possibly influencing their conceptions of leadership [48, 49]. Third, due to rising demands and limited resources, DoN and head nurses in nursing homes are confronted with a conflict between management targets and optimal care delivery. In order to cope with these contradictory circumstances they may use avoidance as a survival strategy [50]. Consequently, staff may perceive absent, passive-avoidant leadership. However, in previous research leadership was described as *flexible, creative* and *supportive* by staff in high-performing nursing homes and as *out-of-touch* in low-performing nursing homes [30]. A recent systematic review confirms the negative influence of passive-avoidant leadership styles such as management by exception and laissez-faire leadership on staff satisfaction with work, job and their leaders, staff health and wellbeing, staff productivity and effectiveness, highlighting the importance of the findings in our study [13].

### Strengths of the study

This study seems unique, because it quantitatively investigates and maps leadership styles and leadership outcomes on different levels in nursing homes. Second, previous research in nursing homes concerning leadership is often limited to the measurement of transformational leadership styles. However, this study includes all leadership styles of the FRLM, enabling a broader insight. Third, the extension of the MLQ-5X concerning leadership outcomes is included, providing insight in results of leadership styles. Fourth, a stringent Bonferroni-adjustment is applied to the alpha-level (*p* < 0.002) to minimize the risk of reporting a statistically significant difference while this is actually not present. Fifth, self-ratings are often used to measure leadership. However, considering the potential influence of leadership on staff performance, in our study, leaders are rated by their staff to avoid self-serving bias through self-ratings [13, 51].

### Limitations of the study

Some limitations warrant further notice. First, the available European Reference Scores (ERS) for the MLQ-5X are not specific for the context of health care. They were derived from higher executive levels of leadership, limiting their suitability for use in nursing homes [21]. Therefore, the comparison of scores between a group of executives (i.e. the ERS) and head nurses in nursing homes should be interpreted with caution. European Reference Scores for leadership in health care settings could support a more accurate comparison. Second, compared with the sample size for raters of head nurses, the small sample size for raters of DoN may limit the generalizability of the results. However, this likely reflects the organizational proportions of functions in nursing home staff. Third, the use of a convenience sample may also limit generalizability. Fourth, the small sample size does not allow comparisons of leadership styles between nursing homes, hindering further subgroup analysis. Fifth, in this descriptive study participants rated leadership styles of their direct leader using the well-established MLQ-5X questionnaire [21]. More nursing home outcome data would need to be evaluated to make more declarative statements of the value of different leadership styles and outcomes. Furthermore, additional qualitative data could have provided a deeper understanding of the results.

### Implications for research

First, although the MLQ-5X visualizes a broad range of leadership styles, reliability analysis reveal poor Cronbach’s alpha values for some subscales. This possibly indicates unsuitability of conceptualizations for the relevant items. Therefore, future research should focus on the development of an instrument specifically designed to measure leadership in the context of nursing homes. Second, given our results, future research should focus on developing interventions to convert passive-avoidant leadership styles to transformational styles on different levels within nursing homes.

### Implications for practice

The presence of passive-avoidant leadership styles in nursing homes may negatively influence their resilience to face the predicted daunting challenges. On the one hand, the constraints present in nursing homes may hinder the development of well-established transformational leadership in head nurses and DoN. On the other hand, passive-avoidant and transactional leadership styles are unlikely to meet the complex demands in this context. The well-known paradox of meeting more needs with fewer resources makes the necessity for effective interventions on leadership and leadership development in nursing homes undeniable. Effective interventions specific to nursing homes currently seem to be lacking, however the participation of head nurses and DoN in existing programs on leadership development could be an important first step.

## Conclusions

Our study describes that passive-avoidant leadership styles are excessively present in contrast to transformational leadership styles in nursing homes. Given the importance of leadership to face current and future challenges, these findings indicate an urgent need to invest in leadership development in nursing homes. To promote transformational leadership, future research should focus on interventions for leadership development. Prior to this, an instrument to measure leadership in the specific context of nursing homes should be developed. This instrument could in turn support the development of transformational leadership in nursing homes.

## Data Availability

The datasets used and/or analysed during the current study are available from the corresponding author on reasonable request.

## Abbreviations

DoN: Director of Nursing
ERS: European Reference Scores
FRLM: Full Range of Leadership Model
MLQ-5X: Multifactor Leadership Questionnaire 5X
NH: Nursing home
